# Photon counting computed tomography in head and neck squamous cell carcinoma: iodine concentration and histopathological features

**DOI:** 10.1007/s00432-026-06509-6

**Published:** 2026-06-01

**Authors:** Alexey Surov, Raihanatou Diallo-Danebrock, Hagen Sonnemann, Ruth Roggel, Martin Scheer, Stefan Volkenstein, Andreas Wienke, Jan Borggrefe

**Affiliations:** 1 Department of Radiology, Neuroradiology and Nuclear Medicine, Johannes Wesling University Hospital, Ruhr University Bochum, Bochum, Germany; 2 Department of Pathology, Johannes Wesling University Hospital Minden, Ruhr University Bochum, Hans-Nolte-Str. 1, 32429 Minden, Germany; 3 Institute of Medical Epidemiology, Biometry and Informatics, University of Halle, Halle, Germany; 4 Department of Oral, Maxillofacial and Plastic Surgery, Johannes Wesling University Hospital, Ruhr University Bochum, Bochum, Germany; 5 Department of Otorhinolaryngology, Head & Neck Surgery, Johannes Wesling University Hospital, Ruhr University Bochum, Bochum, Germany

**Keywords:** Head and neck squamous cell carcinoma, HPV, Photon-counting computed tomography, Iodine concentration, Tumor cellularity

## Abstract

**Purpose:**

Head and neck squamous cell carcinoma (HNSCC) is a common malignancy with increasing relevance in oncologic imaging. Photon-counting computed tomography (PCCT) allows for quantitative assessment in iodine concentration (IC) offering new insights into tumor biology. This study aimed to investigate the association between normalized IC (NIC) and histopathological features in HNSCC.

**Methods:**

Eighty-four patients with primary untreated HNSCC underwent contrast-enhanced PCCT of the neck in venous phase. Iodine maps were generated to quantify intratumoral IC. NIC was calculated as the ratio of tumoral IC to aortal IC. Histopathological analysis included tumor stage, tumor grade, Ki 67 proliferation index, tumor cell count and human papillomavirus (HPV) status. Group comparison were conducted using Mann-Whitney-U tests. Interreader agreement was assessed via intraclass correlation coefficient (ICC). Receiver operating characteristic (ROC) analysis was performed to evaluate diagnostic performance of NIC for HPV status.

**Results:**

NIC values demonstrated excellent interreader reliability (ICC = 0.96, 95%CI = (0.92; 0.98), *p* < 0.01). NIC showed an inverse correlation with tumoral cell count (*r* = -0.24, *p* = 0.03). Tumors with lymphonodal metastases exhibited lower NIC values compared to N0 tumors (0.42 ± 0.20 vs. 0.54 ± 0.26, *p* = 0.02). Cell-rich tumors had lower NIC values than cell-poor tumors (0.42 ± 0.18 vs. 0.53 ± 0.26, *p* = 0.003). HPV negative tumors showed higher NIC values than HPV-positive tumors (0.51 ± 0.23 vs. 0.33 ± 0.18, respectively, *p* < 0.01). A NIC threshold of ≥ 0.5 predicted HPV negative tumors, demonstrating 54.5% sensitivity and 94.4% specificity, with an AUC of 0.74 (95% CI = 0.62–0.86, *p* < 0.01).

**Conclusions:**

NIC derived from PCCT is a robust and reproducible imaging parameter in HSNCC. It is moderately associated with tumor cellularity, nodal involvement, and HPV status. Thus, NIC may serve as a valuable adjunct in non-invasive tumor characterization.

## Introduction

Head and neck squamous cell carcinoma (HNSCC) ranks among the most prevalent malignancies worldwide and remains a major contributor to cancer-related morbidity and mortality (Siegel et al. 2025). Radiological imaging plays a pivotal role, not only in tumor staging but also in providing insights into tumor biology. In recent years, multi-parametric imaging approaches, using computed tomography and particularly magnetic resonance imaging have demonstrated promising associations between imaging biomarkers and histopathological features (Surov et al. 2018; van der Hulst et al. 2023). For instance, several magnetic resonance imaging parameters like apparent diffusion coefficient (ADC) can reflect different histopathological features in HNSCC, such as tumor cell count, proliferation index Ki 67 and expression of p53 (Surov et al. 2018; van der Hulst et al. 2023).

Building on this paradigm, Dual-energy computed tomography (DECT) as well as photon-counting computed tomography (PCCT) introduced new possibilities for oncological staging in the last decade (Johnson 2012; Wu et al. 2023a, b). PCCT emerged as a next generation imaging modality with advantages over conventional CT including improved spatial resolution, reduced electronic noise, and enhanced material decomposition (Wu et al. 2023a, b; Wrazidlo et al. 2023). A key innovation of both dual DECT and PCCT is the ability to quantify tissue iodine concentration (IC), a surrogate marker for perfusion and vascularization, especially in tumors (Surov et al. 2024; Wu et al. 2023a, b; Fan et al. 2017). This possibility presents new opportunities for tumor characterization. Previous studies have shown that IC can distinguish between benign and malignant lesions throughout the body, including the head and neck region (Hsu et al. 2021). Thus, it may correlate with tumor aggressiveness. For example, Foust and colleagues found that IC was lower in metastatic lymph nodes (0.96 ± 0.28 mg/mL) than in non-metastatic nodes (1.65 ± 0.38 mg/mL; *P* = 0.002) in squamous cell carcinoma of the oropharynx (Foust et al. 2018).

Furthermore, IC has been explored as a predictive marker for treatment response and microvascular characteristics in other malignancies, such as rectal, lung and gastric cancers (Surov et al. 2024; Wu et al. 2023a, b; Fan et al. 2017; Wei et al. 2022). IC can predict the efficacy of neoadjuvant chemotherapy and provides imaging evidence to assist in treatment decisions for patients with hypopharyngeal carcinoma Wei et al. 2022). In HNSCC, however, data on the relationship between IC and histopathology remains limited.

Given the biological relevance of angiogenesis and perfusion in tumor progression, we hypothesized that IC may reflect specific histopathological findings and help characterize tumor biology in a non-invasive way. Therefore, the objective of this study was to investigate associations between normalized iodine concentration (NIC) derived from PCCT and the key histopathological features in HNSCC such as tumor stage, cellularity and proliferation. Further, we evaluated specifically the HPV status in HNSCC as a key end-point.

## Materials and methods

### Study design, patients and tumors

**Fig. 1:**
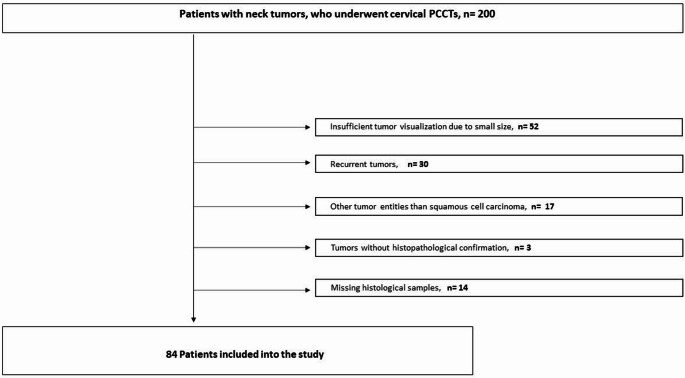
Flow chart of data acquisition

This retrospective, single center study was approved by the institutional review board (Ethics Committee of the Faculty of Medicine, Ruhr-University Bochum, approval code 2021–827) and conducted in accordance with the Declaration of Helsinki. Patients with histologically confirmed primary HNSCC, who underwent contrast-enhanced photon-counting computed tomography (PCCT) between January 2022 and December 2024 were identified from the institutional Picture Archiving and Communication System (PACS). Overall, 200 patients/tumors were identified. Figure 1 gives an overview of the data acquisition.

Inclusion criteria:

- Histopathologically confirmed, previously untreated HNSCC;
- Availability of contrast-enhanced PCCT of the neck region in venous phase;
- available histopathological specimens for analysis.

Exclusion criteria:

- Non-squamous cell tumors such as neuroendocrine tumors, mucinous, and undifferentiated carcinomas;
- Recurrent HNSCC;
- Carcinoma of unknown primary (CUP);
- Relevant imaging artifacts precluding accurate analysis.

Overall, 116 patients/tumors were excluded and the final cohort comprised 84 cases.

Tumor staging (TNM) and grading were obtained from the institutional pathology database.

### PCCT technique

All imaging was performed using a dual-source photon counting CT scanner (NAEOTOM Alpha Prime, software version Syngo CT VA40A/VA50A, Siemens Healthineers, Erlangen, Germany). The scan protocol of the neck in venous contrast phased was acquired with the following parameters: tube voltage, 120 kVp; collimation, 144 × 0.4 mm; pitch factor, 0.8; gantry rotation time, 0.5 s. The tube current was modulated based on the manufacturer-specific dose modulation with set image quality level (IQ level) of 170.

Intravenous contrast medium (ACCUPAQUE^®^ 300, GE Healthcare, Chicago, IL, USA) was administered at a weight-adjusted dose of 1 ml / kg body weight via a coupled automated injector (MEDRAD^®^ Centargo, Bayer Healthcare, Leverkusen, Germany). The contrast medium was applied into a peripheral vein of the arm using a flow rate of 2.4 ml/s followed by 40 ml of saline solution at the same injection rate.

Both polyenergetic and spectral datasets were reconstructed using convolution kernels Br36 (polyenergetic) and Qr36 (spectral data sets), respectively, each using the fourth Quantum Iterative Reconstruction (QIR level 4).

### Imaging analysis

Post-processing and quantitative analysis wer conducted using the vendor specific software (Syngo.Via, software version VB60, Siemens Healthineers, Erlangen, Germany). Two readers independently performed the measurements: One board-certified radiologist with 5 years of experience in head&neck imaging (RR) and one radiologist with 1 year of experience (HS). All measures were performed by the investigators independent to each other and blinded to the histopathological findings.

**Fig. 2:**
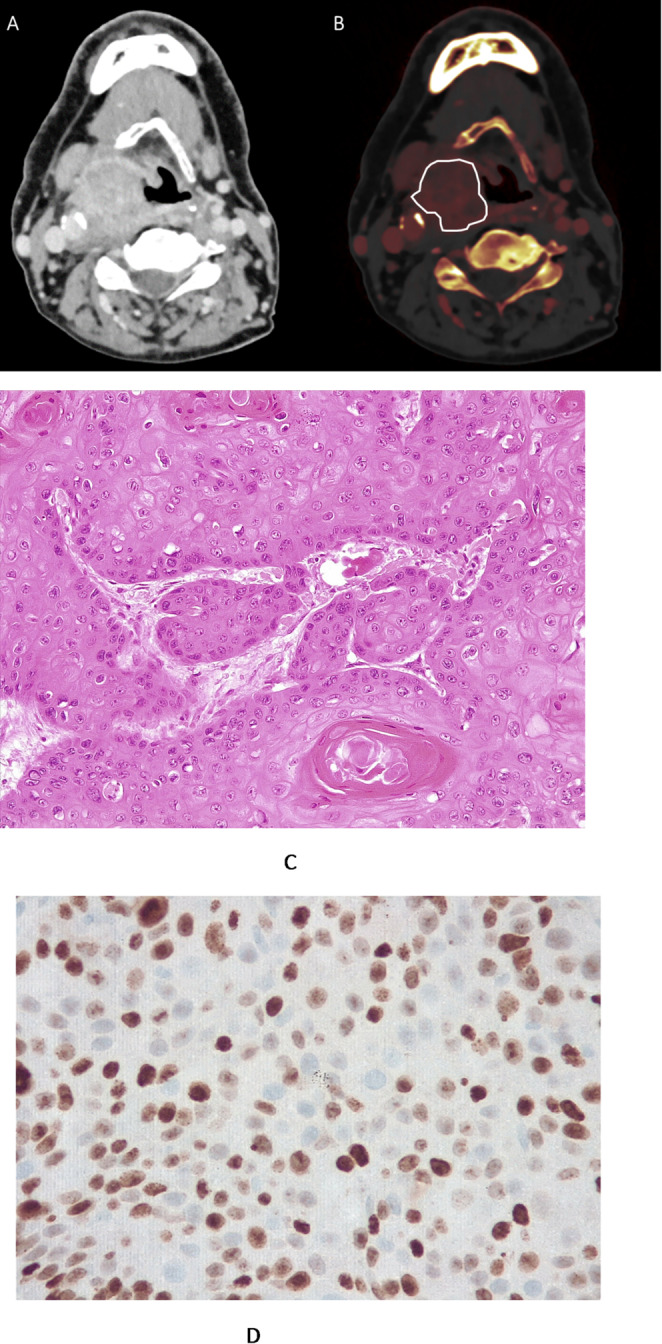
An example of the study cohort. PCCT and pathological findings of a grade 2 T4 N1 M0 HNSCC. **A** CT image showing a large T 4 oropharyngeal tumor. **B** Iodine map with a region of interest around the tumor margins with an IC value of 1.7 mg/mL, NIC value of 0.65. **C** Histological findings (Hematoxilin & eosin stain). Cell count is 249. **D** Histological findings (Immunohistochemical stain, MIB-1 monoclonal antibody). Ki 67-index is 49%

The radiologists underwent task training before data evaluation. In each case, an axial slice with the largest tumor extent of the HSCCM was selected. On this image a polygonal region of interest (ROI) was manually drawn on the iodine map, encompassing the viable tumor while avoiding necrotic, cystic, or hemorrhagic areas as well as vessels (Fig. 2a). For all tumors, mean iodine concentration (IC, mg/ml) was recorded. For normalization, a circular ROI was placed in the ascending aorta to estimate vascular IC. Normalized IC (NIC) was calculated as:

$$\:NIC=\frac{vascular\:IC}{tumoral\:IC}$$

This approach was performed to reduce technical and/or physiological variabilities in iodine accumulation within the tissue of interest due to varying cardiac output and contrast phases (Tian et al. 2022).

### Histopathological analysis

The histological analysis was performed on the bioptic specimens. Tumor specimens were analysed by a board-certified pathologist (RD) with 20 years of clinical experience. Tumor grade was assessed according to the World Health Organization (WHO) criteria (Almangush et al. 2020). Human Papilloma Virus (HPV) status of the tumors was assessed using routine clinical testing using p16 immunohistochemistry (Lewis et al. 2025). The Ki 67 proliferation index was assessed using MIB-1 monoclonal antibody (DakoCytomation, Denmark) on formalin-fixed, paraffin embedded sections. Two high power fields (0.20 mm^2^ per field, x200 magnification) with the highest staining density of positive tumor nuclei were selected, and the Ki 67 proliferation index was calculated as:

$$\:Ki-67\:index\:\left(\%\right)=\frac{Number\:of\:positive\:nuclei}{Total\:number\:of\:nuclei}\:\times\:100$$

Tumor cellularity was defined as the average number of tumor cells per two high-power fields (hematoxylin-eosin (H&E) stained images, x200 magnification, 0.2 mm^2^ per field).

### Statistical analysis

All statistical calculations were performed with the SPSS (IBM SPSS Statistics for Windows, version 28.0). Continuous variables are reported as means, medians and standard deviations, categorical variables as absolute and relative frequencies.

Interreader agreement for NIC values was assessed using intraclass correlation coefficient (ICC) ICC estimate and its 95% confidence interval was calculated using SPSS statistical package version 28 (SPSS Inc, Chicago, IL) based on a mean-rating (k = 2), absolute-agreement, 2-way mixed-effects model. Group comparisons of NIC values (e.g., HPV status, nodal stage) were performed by Wilcoxon test. Spearman’s correlation was used to assess associations between NIC and continuous parameters (e.g. Ki 67 index, cell count). Receiver-operating characteristic (ROC) curve analyses derived from logistic regression was performed to assess NIC´s diagnostic accuracy for HPV status. Sensitivity, specificity, area under the curve (AUC), positive predictive value (PPV) and negative predictive value (NPV) were calculated. Because of the exploratory nature of the present study all p-values were interpreted in an exploratory fashion.

## Results

### Tumor characteristics and tumoral NIC

A total of 84 patients (convenience sample) with primary HNSCC met the inclusion criteria (see Fig. 1). Clinical and histopathological characteristics of the cohort are summarized in Table 1. The majority of tumors were classified as grade 3 (61%), and presented at advanced tumor stage T3-T4 (73.7%). Lymph node involvement (N+) was present in 62% of cases, while distant metastases (M1) were rare (6%). Most tumors were HPV negative (79%) and predominantly located in the oropharynx or oral cavity (63%).

**Table 1:** Included patients and tumors

Patients	
Male	63 (75%)
Female	21 (25%)
Age, M ± SD	67.4 ± 9.2
Tumor localisation	
Oropharynx and oral cavity	53 (63%)
Larynx	17 (20%)
Hypopharynx	7 (8%)
Sinonasal region	4 (5%)
Nasopharynx	3 (4%)
Tumor stage	
T1, n(%)	7 (8.3%)
T2, n(%)	15 (17.9%)
T3, n(%)	18 (21.4%)
T4, n(%)	44 (52.4%)
N0, n(%)	32 (38%)
N+, n(%)	52 (62%)
M0, n(%)	79 (94%)
M+, n(%)	5 (6%)
Tumor grade	
Grade 1, n(%)	1 (1%)
Grade 2, n(%)	32 (38%)
Grade 3, n(%)	51 (38%)
HPV+, n(%)	18 (21%)
HPV-, n(%)	66 (79%)
Ki 67, M ± SD	48.3 ± 18.1%
Cell count, M ± SD	314.5 ± 145.2

T, tumor stage; N, nodal stage; M, distant metastases; HPV, human papilloma virus

The mean value of normalized intratumoral iodine concentration (NIC) was 0.47 ± 0.23, (median: 0.48; range: 0.09–1.03). Interreader reliability of NIC values was excellent, with an ICC of 0.96 (95%CI = (0.92; 0.98), *p* < 0.01).

### Associations between NIC and histopathological parameters

Tumor proliferation: NIC showed nearly no correlation with Ki-67 (*r* = 0.08, *p* = 0.46).

Tumor cellularity: An inverse correlation between NIC and tumoral cell count was observed (*r* = -0.24, *p* = 0.03). Tumors with higher cellularity (cell count above the cohort median value) demonstrated lower NIC values than less cellular lesions (0.42 ± 0.18 vs. 0.53 ± 0.26, respectively, *p* = 0.003).

Lymph node metastases: Tumors with lymph node metastases (N+) had lower NIC values compared to N0 tumors (0.42 ± 0.20 vs. 0.54 ± 0.26, respectively, *p* = 0.02).

**Fig. 3:**
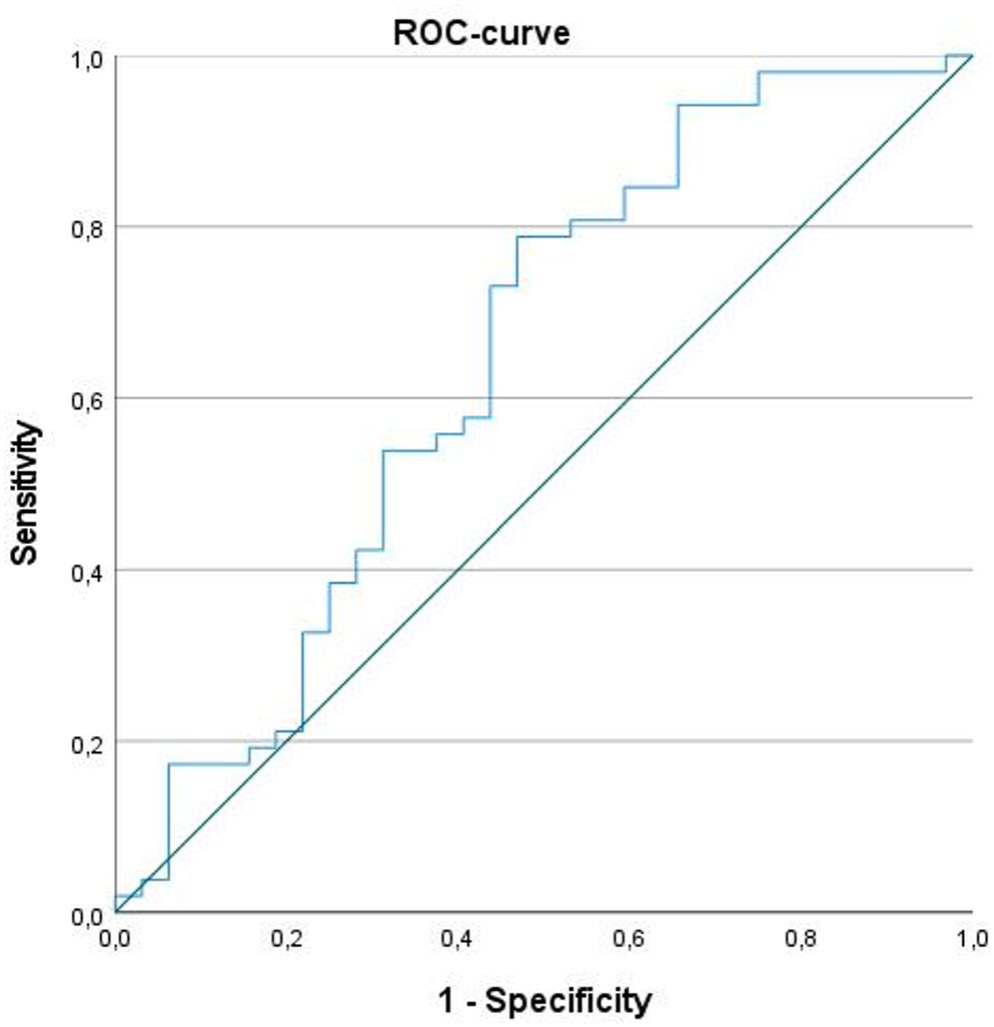
Receiver operating characteristic curve for discrimination of N stage in HNSCC. NIC value of ≥ 0.56 predicts N+ stage (sensitivity = 78.8%, specificity = 53.1%, PPV = 73.2%, NPV = 60.7%, area under the curve = 0.64)

A NIC value of ≥ 0.56 predicted N+ stage with a sensitivity of 78.8%, a specificity of 53.1%, a positive predictive value of 73.2%, a negative predictive value of 60.7%, an area under the curve of 0.64 (95%CI = (0.52; 0.77), *p* = 0.03) (Fig. 3).

Tumor grade or stage: No relevant associations were found between NIC and tumor grade, T stage and M stage (Table 2).

**Table 2:** Comparison of NIC values (mean values ± standard deviation) in HNSCC with different histopathological features

Grade 1/2	Grade 3	*p*-value
0.50 ± 0.25	0.45 ± 0.21	0.26
N0	N+	
0.54 ± 0.26	0.42 ± 0.20	0.02
T1/2	T3/4	
0.49 ± 0.26	0.46 ± 0.22	0.56
M0	M+	
0.47 ± 0.23	0.42 ± 0.16	0.66
HPV-	HPV+	
0.51 ± 0.23	0.33 ± 0.18	0.003
Cell count < median	Cell count > median	
0.53 ± 0.26	0.42 ± 0.18	0.03
Ki67 < median	Ki67 > median	
0.46 ± 0.23	0.49 ± 0.23	0.58

T, tumor stage; N, nodal stage; M, distant metastases; HPV, human papilloma virus; NIC, normalized iodine concentration

**Fig. 4:**
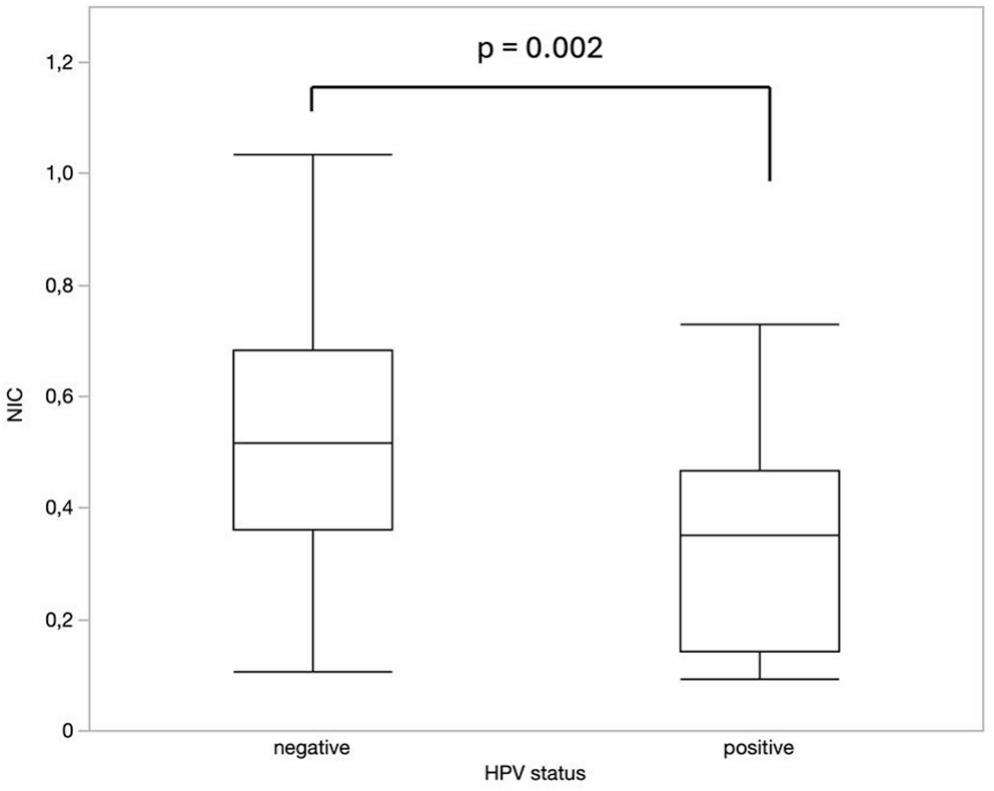
Comparison of NIC values between HPV negative and positive tumors

**Fig. 5:**
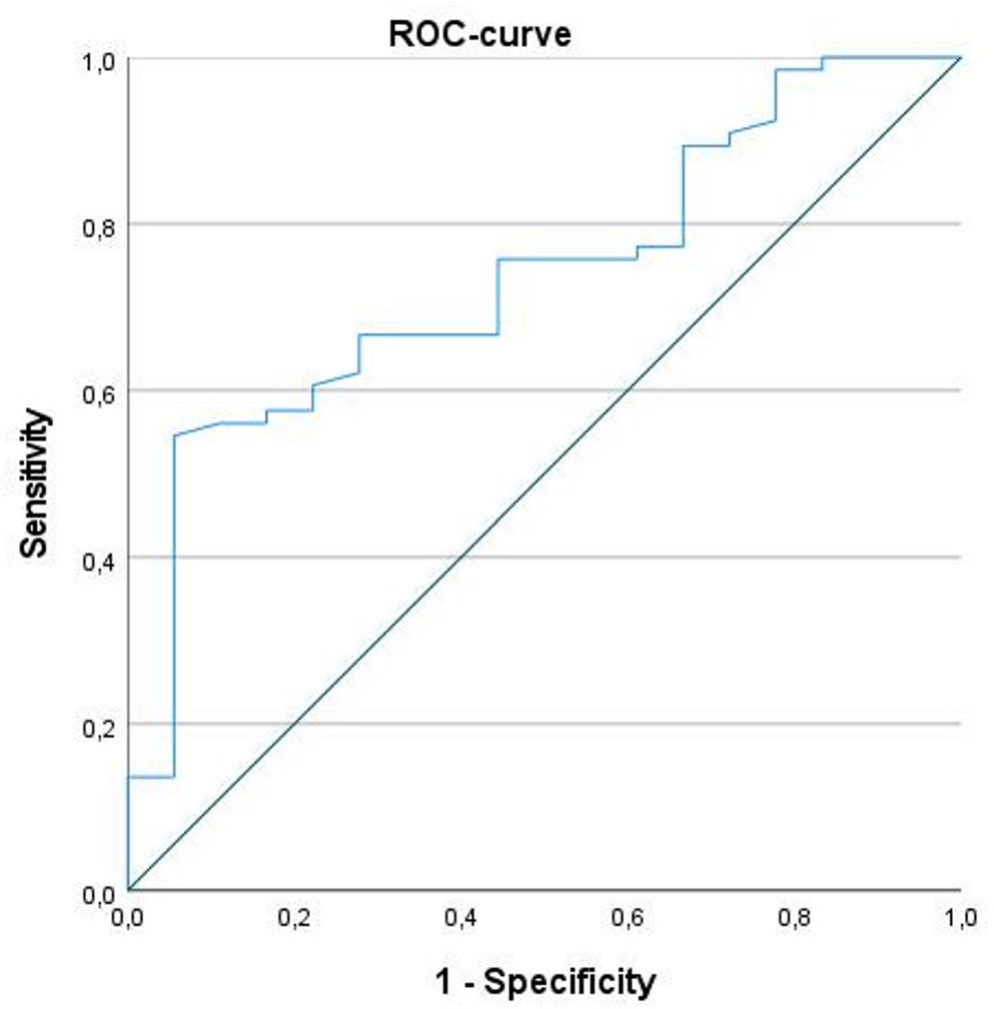
Receiver operating characteristic curve for discrimination of HPV + and HPV- HNSCC. NIC value ≥ 0.5 predicts HPV negative tumors (sensitivity = 54.5%, specificity = 94.4%, PPV = 97.3%, NPV = 36.2%, area under the curve = 0.74

HPV status: HPV negative tumors showed higher NIC values than HPV positive HNSCC, 0.51 ± 0.23 vs. 0.33 ± 0.18, respectively, *p* < 0.003 (Fig. 4). A NIC value of ≥ *0*.5 predicted HPV negative tumors with a sensitivity of 54.5%, a specificity of 94.4%, a positive predictive value of 97.3%, a negative predictive value of 36.2%, an area under the curve of 0.74 (95%CI = 0.62; 0.86, *p* < 0.01) (Fig. 5).

Furthermore, relationships between NIC and histopathology were analyzed in HPV positive and HPV negative tumors separately. In HPV positive HNSCC, tumors with lymph node metastases (N+) showed lower NIC values compared to N0 tumors (Table 3). In HPV positive HNSCC, NIC values were higher in grade 3 lesions in comparison to grade1/2 tumors (Table 4).

**Table 3:** Comparison of NIC values (mean values ± standard deviation) in HPV negative HNSCC with different histopathological features

Grade 1/2	Grade 3	*p*-value
0.55 ± 0.23	0.47 ± 0.23	0.17
N0	N+	
0.58 ± 0.25	0.45 ± 0.20	0.03
T1/2	T3/4	
0.57 ± 0.24	0.48 ± 0.22	0.17
M0	M+	
0.51 ± 0.23	0.44 ± 0.20	0.61
Cell count < median	Cell count > median	
0.56 ± 0.26	0.46 ± 0.17	0.06
Ki67 < median	Ki67 > median	
0.53 ± 0.23	0.49 ± 0.23	0.48

T, tumor stage; N, nodal stage; M, distant metastases; HPV, human papilloma virus; NIC, normalized iodine concentration

**Table 4:** Comparison of NIC values (mean values ± standard deviation) in HPV positive HNSCC with different histopathological features

Grade 1/2	Grade 3	*p*-value
0.16 ± 0.13	0.38 ± 0.16	0.03
N0	N+	
0.27 ± 0.16	0.34 ± 0.18	0.50
T1/2	T3/4	
0.23 ± 0.15	0.37 ± 0.18	0.13
M0	M+	
0.32 ± 0.18	0.40 ± 0.14	0.56
Cell count < median	Cell count > median	
0.37 ± 0.21	0.31 ± 0.16	0.50
Ki67 < median	Ki67 > median	
0.32 ± 0.21	0.34 ± 0.16	0.83

T, tumor stage; N, nodal stage; M, distant metastases; HPV, human papilloma virus; NIC, normalized iodine concentration

## Discussion

This study investigated the association between normalized iodine concentration (NIC derived from photon-counting CT (PCCT) and key histopathological features in head and neck squamous cell carcinoma (HNSCC). Our results demonstrate that NIC is a highly reproducible imaging parameter, with excellent interreader agreement. Importantly, NIC was moderately associated with HPV status. Also NIC showed relationships with nodal stage and tumoral cellularity. This finding indicates that NIC may be used as an additional parameter to characterize HSNCC non-invasively.

To date, most of the existing evidence stems from MRI-based studies. Previous studies have shown that MRI-based parameters such as apparent diffusion coefficient (ADC) and dynamic contrast enhanced (DCE) perfusion metrics correlate well with tumor cellularity, proliferation index (KI-67) (van der Hulst et al. 2023; Swartz et al. 2018; Surov et al. 2016, 2017; Driessen et al. 2016; Nakahira et al. 2014; Jansen et al. 2012). Furthermore, some reports indicated that ADC may discriminate HPV positive and negative tumors (van der Hulst et al. 2023; Driessen et al. 2016). As reported previously, HPV-positive tumors consistently exhibited lower ADC values than HPV-negative tumors (van der Hulst et al. 2023; Driessen et al. 2016; Nakahira et al. 2014). So far, several DCE MRI parameters correlated well with microvessel density and the proliferation marker Ki 67 Surov et al. 2017; Jansen et al. 2012). Finally, positron emission tomography (PET)- CT metrics such as standardized uptake value (SUV) and/or metabolic tumor volume have also demonstrated correlations with microvessel density and tumor in HNSCC (Surov et al. 2019).

However, while CT remains the primary imaging modality for head and neck tumor staging (Gage et al. 2017), its ability to reflect underlying histopathology has traditionally been limited. Previously, associations between CT imaging and histopathology in HNSCC were only demonstrated using complex post-processing imaging analyses such as radiomics feature extraction that are currently neither implemented in the clinical routine nor easy to standardize (Zheng et al. 2023). Only recently, with the advent of DECT and PCCT, has it become possible to extract functional and quantitative imaging data such as iodine concentration, that may reflect tumor vascularity of perfusion and are available in every radiological reading (Wu et al. 2023a, b; Fan et al. 2017). Ever since, there is a growing interest to identify CT derived imaging biomarkers and analyzed as a possible imaging biomarker in different tumors.

Our findings are consistent with recent PCCT and DECT studies in other tumor entities. So far, IC was shown to be associated with lymphovascular invasion in rectal cancer (Surov et al. 2024). Furthermore, IC correlated with expression of Ki 67 in lung and rectal cancers (Wu et al. 2023a, b). In rectal cancer, IC correlated well also with hypoxia-inducible factor 1α (HIF-1α) (Fan et al. 2017). In gastric cancer, IC was associated with microvessel density (Chen et al. 2017).

In HNSCC specifically, only two studies have explored similar questions (Wang et al. 2021; Geng et al. 2023). Wang et al. reported a moderate correlation between IC and Ki 67 in laryngeal cancer. Geng et al. demonstrated that arterial NIC values differed by tumor differentiation grade. Our study builds upon these results and is, to our knowledge, the first to link NIC to HPV status and tumor cellularity in a clinically applicable PCCT setting. Furthermore, our study revealed that the associations between NIC and histopathology were different in HPV positive and HPV negative tumors. This finding may be related to the different tumoral architecture in HPV positive and HPV negative lesions.

We observed that HPV-negative tumors had higher NIC-values that HPV-positive tumors. Of dedicated clinical interest is, that a NIC ≥ 0.5 was, despite it‘s limited sensitivity, highly specific for HPV negative tumors. Furthermore, the study results show that more aggressive tumors had lower NIC values. Prior studies have shown that HPV negative tumors express higher levels of pro-angiogenic markers, such as vascular endothelial growth factor (VEGF), and exhibit increased microvascular density (Baruah et al. 2015; Dok et al. 2017). This could lead to greater iodine accumulation and thus higher NIC values in HPV-negative lesions.

Interistingly, NIC correlated inversely with tumor cell count, supporting the hypothesis that hypercellular tumors may exhibit relatively reduced perfusion due to poor vascular supply or central necrosis (Zhu et al. 2014; Miles 1999; Dou et al. 2020). The observed association between lower NIC values and lymph node metastasis further supports the link between perfusion and tumor aggressiveness.

No relevant association was found between NIC and expression of Ki 67 in our study. This may be due to the complex, non-linear relationship between proliferation and perfusion, or to sampling variability in Ki-67. Furthermore, in the present study, NIC did not reflect tumor grade in HNSCC. This finding is in agreement with the literature. Similarly, Geng et al. did not find any relevant association between venous NIC and tumor stage/grade in HNSCC (Geng et al. 2023).

The ability to non-invasively estimate tumor cellularity or predict HPV status using PCCT could have meaningful clinical implications. HPV negative HNSCC is known to be associated with poorer treatment response, especially to radiotherapy, as well as reduced survival (Park et al. 2025). While biopsy and immunohistochemistry remain the gold standard, NIC could serve as a supplementary marker, particularly in cases where histologiccal confirmation is delayed or challenging. The high specificity of NIC (94.4%) for HPV-negative tumors may make it a valuable tool for treatment planning and stratification in clinical trials. Compared to MRI or PET, PCCT offers the advantage of being rapidly available in routine staging workflows, without the need for time-consuming post-processing or contrast protocols beyond standard practice.

This study has several limitations. First, its retrospective nature intrudes inherent selection bias. Second, our results are based on a relatively small sample size. Third, our histopathological analysis focused on a limited number of parameters; more comprehensive immune or vascular profiling could offer deeper mechanistic insights. We also used p16 staining and it is a surrogate marker for the HPV status. Selection bias may also be present in the histopathological analysis. Finally single-slice tumor sampling may not fully capture intratumoral heterogeneity.

Despite these limitations, NIC is a promising imaging biomarker in HNSCC. Future prospective studies should evaluate its role in larger multicenter cohorts and explore its integration with multiparametric imaging approaches (e.g. PCCT + PET + MRI). Moreover, correlation with outcome data could establish NIC as a prognostic marker.

In conclusion, NIC derived from PCCT is a reproducible parameter. It is associated moderately with tumor cellularity, nodal stage as well as HPV status in HNSCC. It may offer added value in non-invasive tumor characterization and risk stratification, especially in cases where histopathological information is limited or unavailable.

## Data Availability

No datasets were generated or analysed during the current study.

## Declarations

### Guarantor

The scientific guarantor of this study is Alexey Surov.

### Statistics and biometry

One author has statistical expertise (Andreas Wienke).

### Study subjects or cohorts overlap

No study subjects or cohorts have been previously reported.
